# Monitoring of Lassa virus infection in suspected and confirmed cases in Ondo State, Nigeria

**DOI:** 10.11604/pamj.2020.36.253.22104

**Published:** 2020-08-06

**Authors:** Olumuyiwa Babalola Salu, Olufemi Samuel Amoo, Joseph Ojonugwa Shaibu, Chukwuyem Abejegah, Oluwafemi Ayodeji, Adesola Zaidat Musa, Ifeoma Idigbe, Oliver Chukwujekwu Ezechi, Rosemary Ajuma Audu, Babatunde Lawal Salako, Sunday Aremu Omilabu

**Affiliations:** 1 Centre for Human and Zoonotic Virology, Central Research Laboratory, College of Medicine, University of Lagos, Idi-araba, Lagos, Nigeria,; 2 Center for Human Virology and Genomics, Department of Microbiology, Nigerian Institute of Medical Research, Yaba, Lagos, Nigeria,; 3 Infection Control Centre, Federal Medical Centre, Owo, Ondo State, Nigeria,; 4 Monitoring and Evaluation Unit, Nigerian Institute of Medical Research, Yaba, Lagos, Nigeria,; 5 Clinical Science Department, Nigerian Institute of Medical Research, Yaba, Lagos, Nigeria

**Keywords:** Lassa Virus, Lassa fever, reverse transcription polymerase chain reaction, transmission, Ondo

## Abstract

**Introduction:**

Lassa virus (LASV), the causative agent of Lassa fever (LF), an endemic acute viral haemorrhagic illness in Nigeria, is transmitted by direct contact with the rodent, contaminated food or household items. Person-to-person transmission also occurs and sexual transmission has been reported. Thus, this study investigated the presence of LASV in body fluids of suspected and confirmed cases.

**Methods:**

this was a cross-sectional study between March 2018 and April 2019 involving 112 consenting suspected and post ribavirin confirmed cases attending the Lassa fever treatment center in Ondo State. Whole blood was collected from 57 suspected and 29 confirmed cases. Other samples from confirmed cases were 5 each of High Vaginal Swab (HVS) and seminal fluid; 12 breast milk and 4 urine. All samples were analyzed using reverse transcription-PCR (RT-PCR) targeting the S-gene of LASV.

**Results:**

analysis of whole blood by RT-PCR showed that 1/57 (1.8%) suspected and 1/29 (3.4%) confirmed post ribavirin treated cases were positive. While LASV was detected in 2/5 (40%) post ribavirin treated seminal fluids and 1/11 (8.3%) breast milk. However, LASV was not detected in any of the HVS and urine samples.

**Conclusion:**

the detection of LASV in seminal fluid and breast milk of discharged post ribavirin treated cases suggests its persistence in these fluids of recovering Nigerians. The role of postnatal and sexual transmissions in the perennial outbreak of LF needs to be further evaluated.

## Introduction

Lassa fever (LF) is an acute viral haemorrhagic illness endemic in the West African sub-region caused by Lassa virus (LASV) [1,2]. LASV is an enveloped single stranded RNA virus of the family *Arenaviridae*, genus *Mammarenavirus* transmitted to humans via contact with food or household items contaminated with urine or faeces of multimmamate rodents [1,3]. The natural host of LASV is the African rodent *‘Mastomys natalensis’*, which lives close to human settlements [4]. However, evidences suggest that other rodent species such as the African wood mouse, *‘Hylomyscus pamfi’* captured from Nigeria and the Guinea mouse, *‘Mastomys erythroleucus’* captured from both Nigeria and Guinea may also be hosts for LASV [5]. The multimammate rodent quickly produce a large number of offspring, tends to colonize human settlement increasing the risk of rodent - human contact and is found throughout the west, central and eastern parts of the African continent [6]. Once the rodent becomes a carrier, it will excrete the virus throughout the rest of its lifetime through faeces and urine creating ample opportunity for continued exposure within the environment where these rodents cohabit [7]. LASV nucleotide sequences have been established to cluster into six lineages (I-VI) based on geographical locations within the West African Sub-region [8]. Lineages I, II and III circulate in Nigeria, lineage IV circulates in Guinea, Sierra Leone, Liberia, Mali, and Côte d´Ivoire [9-12]. Lineage V circulates in Mali/Ivory Coast [13] and lineage VI in Togo [8].

The LASV is extremely virulent and highly infectious, affecting about 100,000-500,000 persons annually in West Africa with approximately 5,000 deaths occurring yearly [14]. LF claims more lives than Ebola fever because its incidence is much higher [15]. About 80% of persons infected with the Lassa virus are asymptomatic [16]; but in the remaining 20%, the illness manifests as a febrile illness of variable severity associated with multiple organ dysfunctions with or without hemorrhage. About 15-20% of hospitalized Lassa fever patients die from the illness. Ever since the identification of LASV in Nigeria in 1969, there have been several LF outbreaks in the country with an increasing trend of morbidity/mortality, number of states reporting the infection/disease and case fatality rate (CFR) [17]. In year 2016, 2017, 2018 and as at June 2019, the incidences of LF were reported from 18 to 29 out of the 36 states with CFRs of 12.7%, 9.7%, 24% and 22.6% respectively [17,18]. Since yellow fever virus (YFV) and dengue virus (DENV) are also endemic in Nigeria with the country currently dealing with an active yellow fever virus (YFV) outbreak and this was first reported from Kwara state in 2017. Three hundred and forty-one suspected cases of yellow fever were reported from 16 states in 2017 and six of these states had confirmed cases of yellow fever [19,20].

In September 2019 alone, the NCDC reported 243 suspected cases in 42 LGA´s in five states with 10 presumptive positives and one inconclusive [20]. Active dengue infections have also been documented from different parts of the country such as Maiduguri, Ilorin, Ibadan, Ogbomoso, north-eastern Nigeria in Jos and Ibadan respectively [21-23]. Thus, it is important that every sample of suspected cases of VHFs is screened for YFV and DENV as differentials with LASV. Transmission of LASV from human to human has been established within the communities where the disease is endemic [24]. This also includes contact with the corpse of a LF case [24]. Few reports of nosocomial outbreaks in healthcare settings show a disease risk for healthcare workers, which probably were due to poor infection control practices. These include lack of appropriate personal protective equipment, use of contaminated items, failure to adequately disinfect between patients by hand washing. The poor practices facilitated transmission from patient-to-patient or to care givers resulting in high case fatality among healthcare workers [24,25].

It has been reported that the virus is excreted in urine for 3-9 weeks post infection, remains in the semen for up to 3 months after becoming infected and has also been detected 64 days after patient recovery and discharge from hospital [26,27]. Sexual transmission of LASV has also been reported. However, the extent of this transmission dynamic remains unknown and requires further evaluation in communities where this disease is endemic [16,28]. No study has proven the presence of LASV in breast milk, but the high level of viraemia suggests it may be possible [29]. Although, it was reported that the circulating strains of LASV appear to be very similar to those from previous years in the country [30], the transmission dynamics of this virus particularly in endemic regions are yet to be fully understood. Even though there is a risk for sexual and/or post-natal transmission of LASV, the persistence of LASV in semen, urine and other body fluids of Nigerians has not been reported. Therefore, this study investigated the presence of LASV, as well as YFV and DENV as differentials, in body fluids of suspected, confirmed and discharged cases to better understand its role in the perennial outbreaks of LF in Nigeria.

## Methods

**Figure 1:**
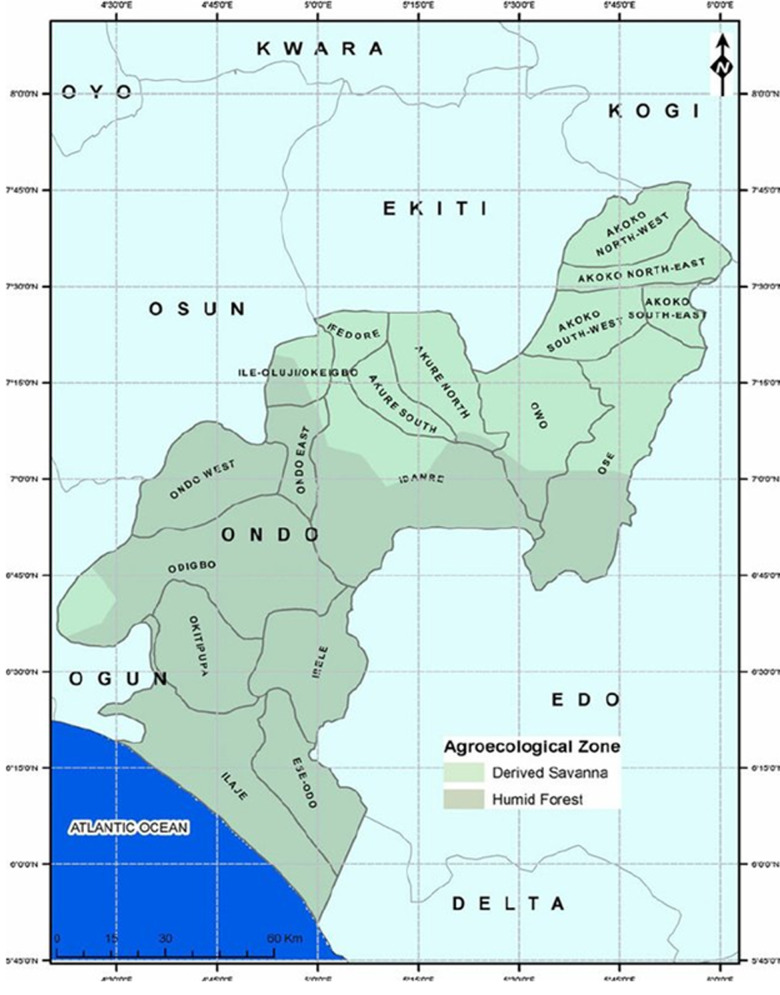
map of Ondo State (study area) showing the two eco-climatic zones, local government areas and bordering states

**Study location/area:** this study was carried out in Ondo State, Nigeria. The state has Akure as its state capital with eighteen local government areas, the major ones being Akoko, Akure, Okitipupa, Ondo and Owo. Ondo State borders Ekiti state to the north, Kogi State to the northeast, Edo State to the east, Delta State to the southeast, Ogun State to the southwest and Osun State to the northwest as highlighted in Figure 1 [31]. Ondo State has had repeated outbreaks with confirmed cases of Lassa fever within the study period and confirmed cases from other states had also been traced to the state. All suspected cases from the different Local Government Areas (LGAs) within the state, are referred to the Lassa fever treatment center at the Federal Medical Center (FMC), Owo for diagnosis and management.

**Study design and population:** this was a cross-sectional study conducted between March 2018 and April 2019 with a total of 112 consenting individuals. These individuals comprised of 57(25.9%) suspected and 29(25.9%) confirmed cases who were still on admission at the Lassa fever treatment center at the Federal Medical Center (FMC), Owo, Ondo State. Additional 26 individuals (23.2%) were outpatients living in the communities who had previously been treated with ribavirin and discharged from the Lassa fever treatment center but were still attending the hospital for post discharge evaluations.

**Informed consent/ethical approval:** an ethical approval was obtained from the Institutional Review Board (IRB) of the Nigerian Institute of Medical Research (NIMR). Furthermore, approval was also obtained from the Ondo State Ministry of Health before commencement of study. Only individuals who had given prior documented consent were included in this study.

**Specimen collection, transportation, handling and processing:** the total number of one hundred and twelve (112) consenting individuals who participated in this study comprised of 66(58.9%) males and 46(41.1%) females with a mean age of 42.6 years (SD ± 6.8). Whole blood samples were collected from 57 suspected cases and 29 confirmed cases of Lassa fever. Other samples collected from discharged cases include 5 high vaginal swab (HVS) and seminal fluid each; 12 breast milk and 4 urine samples. All samples were packaged in triplicates and transported in cold-chain from Ondo State to the Centre for Human and Zoonotic Virology (CHAZVY), Central Research Laboratory, College of Medicine of the University of Lagos. Universal sample precautions and handling procedures were carried out as recommended by the United States Centers for Disease Control and Prevention [32]. All specimen transport containers were disinfected with 10% hypochlorite solution in an airtight glove box. Viral agents in specimen aliquots (undiluted and 1: 10 dilution) were inactivated in guanidinium-thiocyanate-based lysis buffer at room temperature for 10 minutes before extraction of viral nucleic acid.

**Nucleic acid extraction and reverse transcriptase-polymerase chain reaction:** the viral nucleic acid from inactivated sample aliquots (undiluted and 1: 10 dilution) were extracted using a mini spin column RNA extraction kit by Qiagen (Qiagen, Germantown, Maryland, United States) in a class IIA biological safety cabinet according to the manufacturer´s instructions. After the extraction of viral nucleic acid, S segment of the RNA genome, 3´ and 5´ non-coding regions of the nucleic acid of LASV, dengue and yellow fever viruses were amplified in discrete RT-PCRs with primers as listed in Table 1. Separate reaction mixtures for Lassa, dengue and yellow fever viruses were prepared and cycled as described in the one-step RT-PCR kit by AmbionAgPath-ID protocol (Applied Bio-systems, Foster City, California, United States). The reaction was performed using the 9700 applied bio-systems thermal cycler with the following temperature profile: 50°C for 30 min and 95°C for 5 min, followed by 35 cycles of 95°C for 30 s, 55°C for 30s, and 72°C for 30s with a final extension of 72°C for 5 min. Subsequently, PCR amplicons were subjected to 1.5% agarose gel electrophoresis with 1X SYBR® safe DNA gel staining dye (Invitrogen, Carlsbad, California, United States) for 30 min at 120V/400mA and images of amplicon bands under UV light were taken with BioDocAnalyze 2.0 (Biometra, Goettingen, Germany). The positive control used for Lassa assays were previously detected Lassa samples from Irrua, Edo State, Nigeria with accession number GU481078 NIG 08-A47 2008 IRRUA, while those for dengue and YFV assays were both tissue culture inactivated samples both from the Virology Unit Laboratory of the Bernhard Nocht Institute of Tropical Medicine, Hamburg, Germany through our collaborations.

**Table 1:** primers used for Lassa, dengue and yellow fevers investigation

Virus	Primer Name	Primer Sequence	Amplicon Size Base Pair (bp)
Lassa fever virus	36E2	5´GTT CTT TGT GCA GGA MAG GGG CAT KGT CAT 3´	~320
	LVS-339-rev	5´ ACC GGG GAT CCTAGG CAT TT 3´	
Dengue fever virus	DenS	5´GGA TAG ACC AGA GAT CCT GCT GT 3´	79
	DenAs	5´ CAT TCC ATT TTC TGG CGT TC 3´	
	DenAs+	5´ CAG CAT CAT TCC AGG CAC AG 3´	
Yellow fever virus (in-house)	YF fwd	5´ ATG GCA CTG TTG TGA TGC AG 3´	405
	YF rvs	5´ AGT TCA AGC CGC CAA ATA GC 3´	

## Results

**Figure 2:**
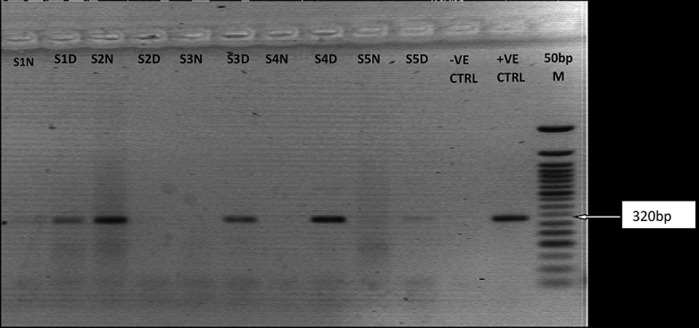
RT-PCR Detection of S-Gene fragment of Lassa virus. The gel lanes represent neat (undiluted, N) and 1: 10 dilutions (D) of the RNA extracts used for RTPCR. Lassa positive samples were represented in lanes S1-S5. RNase/DNase free water was used as negative extraction/RTPCR control (-ve CTRL) while a 2008 outbreak positive sample (GU481078_NIG_08-A47_2008_IRRUA) was used as positive control (+ve CTRL)

**Figure 3:**
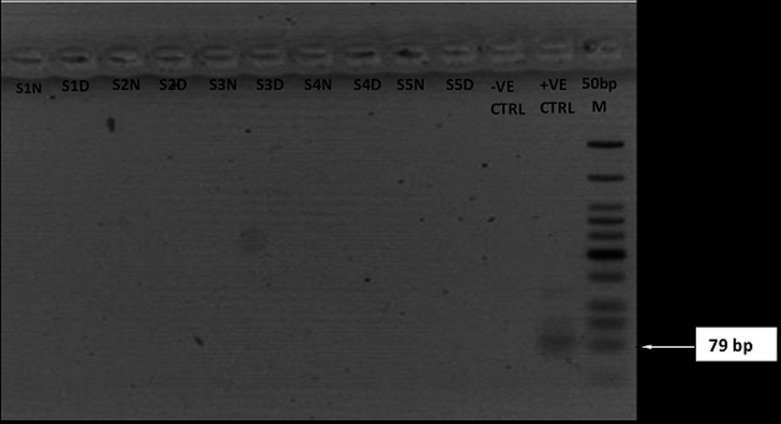
RT-PCR detection of dengue virus. The gel lanes represent neat (undiluted, N) and 1: 10 dilutions (D) of the RNA extracts used for RTPCR. Dengue negative samples were represented in lanes S1-S5. RNase/DNase free water was used as negative extraction/RTPCR control (-ve CTRL) while a tissue culture inactivated sample of dengue virus from the virology unit laboratory of the Bernhard Nocht Institute of Tropical Medicine, Germany was used as positive control (+ve CTRL)

**Figure 4:**
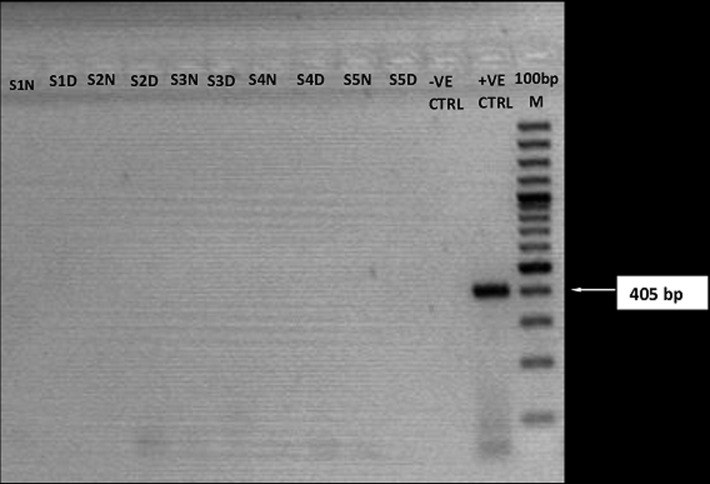
RT-PCR detection of yellow fever virus. The gel lanes represent neat (undiluted, N) and 1:10 dilutions (D) of the RNA extracts used for RTPCR. Yellow fever negative samples were represented in lanes S1-S5. RNase/DNase free water was used as negative extraction/RTPCR control (-ve CTRL) while a tissue culture inactivated sample of 17D yellow fever strain from the virology unit laboratory of the Bernhard Nocht Institute of Tropical Medicine, Germany was used as positive control (+ve CTRL)

**Reverse transcriptase-polymerase chain reaction amplification and agarose gel analysis of Lassa, yellow fever and dengue viruses:** all samples collected were analyzed for LASV RNA by RT-PCR. The expected amplicon band size of approximately 320 base pairs (bp) of the S segment of the RNA genome for LASV was detected by the agarose gel electrophoresis analysis (Figure 2). The detected band size of the LASV amplicons was at par with the positive LASV controls and no band was observed in the negative control lane on the gel picture (PCR-grade water) (Figure 2). However, none of the expected band sizes (~79bp) and (~405bp) were detected for dengue viruses or yellow fever (Figure 3,Figure 4). Analysis of whole blood by RT-PCR showed that 1/57(1.8%) suspected and 1/29(3.4%) confirmed ribavirin treated case still on admission at FMC, Owo were positive for LASV (Table 2). While LASV was still detected in 1/11(8.3%) breast milk and 2/5(40%) post ribavirin treated seminal fluids from discharged individuals in the community. However, LASV was not detected in any of the HVS and urine samples (Table 2).

**Table 2:** distribution of LASV positivity by category of cases

Categories of Cases	Sample Type	Total Number	RT-PCR Positive	%
Suspected (on admission)	Whole Blood	57	1	1.8
Confirmed (on admission)		29	1	3.4
Discharged (in community)	Breast Milk	12	1	8.3
	Seminal Fluids	5	2	40
	High Vaginal Swab	5	0	0
	Urine	4	0	0

## Discussion

Large scale outbreaks of LF have been reported in Nigeria since 2015 with the disease occurring all year round. The main preventive strategies in endemic areas are: effective rodent control in areas close to living environment; avoiding contacts with rodents and the consumption of it. Others include high index of suspicion for LF; prompt referral from such health facilities to treatment centers and proper use of infection control practices in health facilities [33-35]. Although LASV is found in virtually all body fluids and compartment during acute infection, little is known about the viral kinetics of LASV in body fluids other than blood after recovery from the disease [36]. The burden of the disease and the role of transmission through other body fluids other than blood after recovery still remains a puzzle in the country. The non-detection of both YFV and DENV from this set of suspected, confirmed and discharged cases of VHFs in Ondo State, implies that Lassa virus remains the major agent and driver of VHFs in the country. However, since there have been reported cases of YFV and DENV with documented evidences that these viruses are all endemic in the country, testing for these agents alongside with LASV should remain a continuum particularly as it has been documented that only 20% of the cases of VHF are confirmed to be caused by LASV [16]. The detection of LASV in seminal fluid and breast milk of discharged post ribavirin treated cases and its persistence in these fluids of recovering individuals has been established in this study as documented in previous studies.

The presence of LASV in the body fluids of discharged cases is useful for clinical and public health reasons in Nigeria. This is particularly important because of the high mortality and morbidity rates in addition to its perennial nature. Thus, counseling on the possibilities of LASV transmission from men after discharge from treatment centres to their sexual partners and from mothers to their offspring during breast feeding is strongly advocated. Safe sex practices including sexual abstinence and use of male or female latex condom as well as abstinence from breast feeding by nursing mothers after discharge should be encouraged among survivors. The implications of viral persistence in such immune sanctuaries are now being recognized as potential sources of new outbreaks through sexual transmission for a number of other emerging infectious viruses, including Lassa, ebola and Zika viruses [36-39]. Therefore, the identification of viral persistence in seminal fluid and breast milk draws critical attention to the need to follow LF survivors´ longitudinally after clearance of viremia. Thus, survivors should also be offered access to care and prevention, particularly with the possibility to test other body fluid other than blood after discharge from any LF treatment centre which provides them with possibilities to mitigate any risks that may occur. The testing of other body fluids of LF survivors should be considered as a possibility to be included for improved LF management and treatment network coordinated by the Nigerian Centre for Disease Control (NCDC).

However, given these findings, the following questions still need to be addressed; are the LASV shed in seminal fluid and breast milk viable and infective, for how long and at what concentrations? It has been over 50 years since LASV was first identified during a 1969 outbreak in Nigeria, but the mechanisms underlying its pathogenesis and the role that host and viral factors play in the transmission dynamics and persistent outbreaks remain unclear. The answers to these questions have implications for ascertaining the risks for sexual and post-natal transmission and therefore, its effects on epidemiologic and transmission dynamics. Although, LASV has been detected in human semen and other body fluids, the extent to which LASV existence and replication occurs within these fluids remains unclear [34,35]. The shedding of LASV in the urine for 3 to 6 weeks and up to 3 months in seminal fluid, with risk for sexual transmission, prompting condom use in survivors had been well documented [26,28,40,41]. Though a previous study had no proven evidence of the presence of LASV in breast milk [29], this study documents a proven evidence of the detection of LASV in breast milk of mothers who had recovered from LF disease. This study therefore puts to rest the speculations on the detection of this virus in breast milk. Moreover, this study was limited by the small numbers of post ribavirin treated individuals who recovered and were discharged. Thus, the actual duration of shedding of LASV in the seminal fluid and breast milk of these individuals could not be ascertained with this study.

## Conclusion

Lassa fever outbreak remains a public health threat and it is a burden on the vulnerable population within and outside the endemic states in Nigeria. Thus, the continued monitoring of individuals that are discharged from the treatment centres in Nigeria and the role of postnatal and sexual transmissions in the perennial outbreak of LF in Nigeria needs to be further evaluated.

### What is known about this topic

- Several Lassa fever outbreaks had been witnessed in Nigeria since the discovery of the Lassa virus in 1969;
- There has been an increasing trend of morbidity/mortality, number of states reporting the infection/disease and case fatality rate (CFR) since year 2016;
- The natural host of LASV is the multimammate African rodent ‘Mastomys natalensis’, which lives close to human settlement and transmitted to humans via contact with food or household items contaminated with urine or faeces of this rodent.

### What this study adds

- Data presented here established and confirmed the detection and persistence of LASV RNA in the seminal fluid and breast milk of discharged post ribavirin treated Lassa fever cases in Nigeria;
- The detection and persistence of LASV RNA in these body fluids of discharged cases in Nigeria is useful for clinical and public health reasons, based on the high mortality and morbidity rates and the perennial nature of LF in Nigeria;
- This study with the proven evidence of the detection of LASV RNA in breast milk of mothers who had recovered from LF disease, puts to rest the speculations on the detection of this virus in breast milk.
